# Circulating nuclear factor-kappa B mediates cancer-associated inflammation in human breast and colon cancer

**DOI:** 10.5937/jomb0-27128

**Published:** 2021-03-12

**Authors:** Kundaktepe Berrin Papila, Volkan Sozer, Kocael Pinar Cigdem, Sinem Durmus, Dilara Kurtulus, Cigdem Papila, Remise Gelisgen, Hafize Uzun

**Affiliations:** 1 Istanbul University-Cerrahpasa, Faculty of Cerrahpasa Medicine, Department of General Surgery, Istanbul, Turkey; 2 Yildiz Technical University, Department of Biochemistry, Istanbul, Turkey; 3 Istanbul University-Cerrahpasa, Faculty of Cerrahpasa Medicine, Department of Medical Biochemistry, Istanbul, Turkey; 4 Istanbul University-Cerrahpasa, Faculty of Cerrahpasa Medicine, Istanbul, Turkey; 5 Istanbul University-Cerrahpasa, Faculty of Cerrahpasa Medicine, Department of Internal Medicine, Division of Oncology, Istanbul, Turkey

**Keywords:** nuclear factor kappa-B, tumour necrosis factor-α, soluble TNF-related apoptosis-inducing ligand, interleukin-6, pentraxin-3, nuklearni faktor kapa-B, faktor nekroze tumora-α, rastvorljivi ligand vezan za TNF koji indukuje apoptozu, interleukin-6, pentraksin-3

## Abstract

**Background:**

Inflammation is recognized as a hallmark feature of cancer development and progression. The aim of our study was to investigate the significance of serum nuclear factor kappa-B (NF-κB) levels as a circulating marker in the monitoring of inflammation in breast and colon cancer; to show the relationship between NF-κB with inflammatory parameters as tumour necrosis factor-α (TNF-α), soluble TNF-related apoptosis-inducing ligand (sTRAIL), interleukin-6 (IL-6), pentraxin-3 (PTX-3), procalcitonin (PCT), and C-reactive protein (CRP) levels.

**Methods:**

Serum NF-κB, TNF-α, sTRAIL, IL-6, PTX-3, PCT, and serum CRP levels were measured using enzyme-linked immunosorbent assay (ELISA) in 40 patients with breast cancer, 40 patients with colon cancer and 30 healthy controls.

**Results:**

The serum NF-κB, TNF-α, IL-6, PTX-3, PCT, and serum CRP concentration was significantly higher, and the serum sTRAIL concentration was significantly lower in the patients with breast and colon cancer than in healthy controls. NF-κB was positively correlated with CRP and negatively correlated with sTRAIL.

**Conclusions:**

These results suggest that increased NF-κB may decrease the clinical efficacy of sTRAIL in solid tumour cells. There is a relationship between inflammation and carcinogenesis so that the development of cancer occurs with chronic inflammation in breast and colon. The study results have shown that colon and breast cancer patients have increased systemic inflammation, as measured by increased circulating cytokines, and acute-phase proteins, or by abnormalities in circulating cells. NF-κB may combine with other markers of the systemic inflammatory response in prognostic scores in cancer. In addition to surgical resection of the tumour, and conventional radio and chemotherapy for cancer treatment, the use of sTRAIL or other agonists for cancer therapy appeared a new potential therapy.

## Abstract (sr)

**Uvod:**

Upala se smatra karakterističnim svojstvom razvoja i napredovanja raka. Cilj ovog istraživanja je bio da se ispita značaj nivoa nuklearnog faktora kapa-B (NF-kB) u serumu kao cirkulišućeg markera u praćenju upale kod karcinoma dojke i debelog creva; prikazati vezu između NF-kB i inflamatornih parametara kao što su nivoi faktora nekroze tumora-α (TNF-α), rastvorljivog liganda vezanog za TNF koji indukuje apoptozu (sTRAIL), interleukina-6 (IL-6), pentraksina-3 (PTX-3), prokalcitonina (PCT) i C-reaktivnog proteina (CRP).

**Metode:**

Nivoi NF-kB, TNF-α, sTRAIL, IL-6, PTX-3, PCT i CRP u serumu mereni su korišćenjem imunosorbenta vezanog za enzim (ELISA) kod 40 pacijenata sa karcinomom dojke, 40 pacijenata sa karcinomom debelog creva i 30 zdravih osoba u kontrolnoj grupi.

**Rezultati:**

Koncentracija inflamatornih parametara NF-kB, TNF, IL-6, PTX-3, PCT i CRP u serumu bila je značajno veća, a koncentracija sTRAIL u serumu bila je značajno manja kod pacijenata sa karcinomom dojke i debelog creva nego kod kontrolne grupe. NF-kB bio je u pozitivnoj korelaciji sa CRP-om i negativnoj sa sTRAIL-om.

**Zaključak:**

Ovi rezultati sugerišu da povećani NF-kB može umanjiti kliničku efikasnost sTRAIL u čvrstim ćelijama tumora. Postoji veza između upale i karcinogeneze, tako da razvoj karcinoma počinje sa hroničnom upalom dojke i debelog creva. Rezultati istraživanja su pokazali da pacijenti sa karcinomom debelog creva i dojke imaju pojačanu sistemsku upalu, merenu povećanim cirkulišućim citokinima i proteinima akutne faze, ili abnormalnostima u cirkulišućim ćelijama. NF-kB se može kombinovati sa drugim markerima sistemskog inflamatornog odgovora u prognostičkim procenama raka. Pored hirurške resekcije tumora, konvencionalne radioterapije i hemoterapije za lečenje raka, upotreba sTRAIL ili drugih agonista za terapiju raka pojavila se kao nova potencijalna terapija.

## Introduction

Inflammation is part of the complex response of body tissues that occurs in reaction to several types of injury, such as pathogens, damaged cells, and irritants. The inflammatory process keeps the homeostasis balanced and limits itself after the triggered event has been eradicated. It is well known that inflammation takes part in the pathophysiology of some disorders, including cancer. Its role in the cancer types has been studied from the end of the 19 th century and recognised as a hallmark feature of cancer development and pathogenesis [1].

Recent studies focused on the role and effect of certain molecules in the progression of inflammation, such as chemokines, cytokines, transcription factors [2] [3]. The nuclear factor-κB (NF-κB) is one of these transcription factors which play a significant role in the regulation of many physiological processes such as immune responses, cell death, cell survival as well as inflammation [4]. As it is found in all of the cells in the body, it is critical for human health. The disruption of NF-κB function contributes to the activation of various inflammatory pathways which may cause carcino genesis of an otherwise healthy cell. Researches showed editing role in the pathogenesis of cancer and indicated its relation with mediators such as tumour necrosis factor-α (TNF-α), soluble TNF-related apoptosis-inducing ligand (sTRAIL), interleukin-6 (IL-6), pentraxin-3 (PTX-3), procalcitonin (PCT), and Creactive protein (CRP) levels [5] [6] [7] [8] [9] [10] [11]. Each one of these molecules is sensitive to regulation in inflammation. Therefore, they are widely used to monitor the level of inflammation as body fluids.

The aim of our study was to investigate the significance of NF-κB levels as a serum marker in the monitoring of inflammation in breast and colon cancer; to show the relationship between NF-κB with inflammatory parameters. As inflammation is one of the important steps of cancer pathogenesis, it might be hypothesized that serum levels of inflammatory markers reflect the changes in the clinical status of the patient. We are of the opinion that the combination of the new and old parameters related to cancer is important to help the clinical doctors in the correct diagnosis and effective treatment and to give clues about the prognosis of patients. In this respect, our study may support the findings in recent researches and may guide future studies.

## Materials and Methods

This study was conducted at the Department of Internal Medicine, Division of Oncology, and Department of General Surgery, Faculty of Cerrahpasa Medicine, Istanbul University-Cerrahpasa. The protocol for sample collection was approved by the Ethical Committee of the Cerrahpasa Medical Faculty. The study was performed in accordance with the Helsinki Declaration, and informed consent was obtained from all patients and controls prior to their inclusion in the study.

A total of 80 patients were admitted during the period of the study. Thirty criteria-matched healthy individuals were enrolled in this study. The patients, either in the study or control group, who had cardiovascular diseases, diabetes mellitus, renal failure, autoimmune disease, chronic infection and inflammation, alcohol abuse, and those who used anti lipi demic and antioxidant drugs, anti-inflammatory drugs, corticosteroid, immunosuppressive drugs, were excluded from the study. As NF-κB, as well as serum parameters, are strongly related to inflammation in body cells, diseases that have a chronic inflammatory period such as diabetes mellitus, renal diseases, and cardiovascular diseases might deflect the results. Therefore, we have made a questionnaire which included questions on demographics, diets, lifestyle factors. Considering the answers, we have chosen the patients who had similar lifestyles and diets. All of the patients with neoadjuvant treatment were also excluded from the study. Studies suggest that treatment options in cancer may regulate NF-κB activity [12]. The remaining group included 40 patients with breast cancer and 40 patients of colon cancer. Breast cancer patients in the study had distant metastases at the time of diagnosis. We evaluated clinicopathological features (histology, menopausal status, estrogen receptor (ER), progesterone receptor (PR) status, number of axillary lymph nodes involved, grade, tumour size, and stage according to the American Joint Committee on Cancer staging system. The patients with newly diagnosed and histologically confirmed primary colorectal cancer were included in this study. Tumour staging was performed according to the Dukes' and TNM Classification of Malignant Tumours (TNM).

Blood was drawn after 12–14 h of fasting in the morning. Serums were obtained after at least 30 min of clotting by centrifugation at 2,500 g for 15 min. Serums were removed and used directly for measurements of biochemical parameters and tumour markers. Remaining serum was stored at -80°C until assayed for determination of all parameters. All icteric or hemolytic blood samples were discarded. All parameters were analysed in all samples together in a single batch after we had finished our protocol (control and patient samples were analysed in the same batch).

### Measurement of Serum NF-κB Concentrations

The serum NF-κB levels were determined using the commercial human ELISA Kit (Cusabio Biotech, catalogue number: CSB-E12107h) according to the manufacturer's directions. The coefficients of intraand inter-assay variation were 5.3% (n=20) and 6.4% (n=20), respectively.

### Measurement of Serum sTRAIL Concentrations

The serum sTRAIL levels were determined using the commercial human sTRAIL ELISA Kit (Quantikine^®^, R&D Systems, Minneapolis, MN, USA) according to the manufacturer's directions. The coefficients of intra- and inter-assay variation were 5.5% (n=20) and 6.7% (n=20), respectively.

### Measurement of Serum TNF-α Concentrations

The serum TNF-α levels were determined using the commercial human TNF-α ELISA Kit (Quantikine^®^, R&D Systems, Minneapolis, MN, USA) according to the manufacturer's directions. The coefficients of intra- and inter-assay variation were 5.1% (n=20) and 6.1% (n=20), respectively.

### Measurement of serum IL-6 Concentrations

The serum IL-6 levels were determined using the commercial human IL-6 ELISA Kit (Quantikine^®^, R&D Systems, Minneapolis, MN, USA) according to the manufacturer's directions. The coefficients of intra- and inter-assay variation were 5.0% (n=20) and 5.9% (n=20), respectively.

### Measurement of serum PTX-3 Concentrations

The serum PTX-3 levels were determined using the commercial Human PTX-3 ELISA Kit (HumanPentraxin-3, Hycult Biotechnology, HP9039; Uden, The Netherlands) according to the manufacturer's directions. The coefficients of intra- and inter-assay variation were 5.3% (n=20) and 6.1% (n=20), respectively.

### Measurement of plasma PCT concentrations

The plasma PCT levels were determined using the commercial Human PCT ELISA Kit (Uscn Life Science Inc., ELISA Kit, Cat. No: SEA689Hu, USA) according to the manufacturer's directions. The coefficients of intra- and inter-assay variation were 5.0% (n=20) and 5.9% (n=20), respectively.

The serum CRP levels were measured using a nephelometric method (Immage 800 Beckman Coulter).

Tumour markers (CEA, CA19-9, CA15-3) were measured using an IMMULITE 2000 (DPC, Los Angeles, CA). CA15-3 was analysed using the chemiluminescent immunometric assay. CA19-9 and CEA were measured by immunometric assay.

### Statistical analysis

All statistical analyses were carried out using SPSS v. 22.0 (IBM, Armonk, NY, USA) package program. The distribution of all analysed parameters was confirmed using the Kolmogorov-Smirnov test. The 2 test was used for categorical data. For correlation analysis, Spearman's was used. Continuous variables were tested for normal distribution using the Shapiro-Wilk test. Results for normally distributed continuous variables were expressed as means ± standard deviations. Statistical significance of the differences between means was determined by Student's t-test or ANOVA followed by post hoc multiple comparisons using Tukey HSD. Correlations among continuous variables were assessed using Spearman's rank correlation coefficient (r). Categorical variables were expressed as numbers (percentages) and were compared using Fisher's exact test. The receiver operating characteristic (ROC) analysis was used to determine the separation power of the parameters. As a result of the ROC analysis, cut-off points were determined by using the Youden Index. The risk analysis was performed to determine the risk of having the values above the cut-off value, and the OR (odds ratio) values were obtained. Since small numbers increase the estimation bias, the Haldane's correction was used. The p-values < 0.05 were considered statistically significant.

## Results

The demographic features and tumour marker levels of all subjects included in the study are shown in Table 1. No statistically significant difference was found between the groups in terms of age, sex, and WBC counts. CA15.3 and CEA tumour markers were statistically significantly higher in breast cancer patients than in control individuals (respectively p<0.01 and p<0.001). In addition, all of the biochemical parameters we analysed were statistically significant in both breast cancer patients (NF-κB: p<0.001; sTRAIL: p<0.01; TNF-α: p<0.001; IL-6: p<0.001; PCT: p<0.001; PTX-3: p<0.001; CRP: p<0.001) and colon cancer patients (for all p<0.001) compared to the control. In addition, TNF-α, IL-6, PCT, and PTX-3 levels were found statistically significant in colon cancer patients compared to breast cancer patients (for all p<0.001) Table 2 and Table 3 summarize the clinicopathological features of breast cancer and colon cancer patients, respectively.

**Table 1:** Demographic features and tumor marker and biochemical parameters levels in control, breast cancer, and colon cancer cases (mean± SD). BMI, body mass index; CA-15.3, cancer antigen 15.3; CA-19.9, cancer antigen 19.9; CEA, carcinoembryonic antigen; NF-κB, nuclear factor kappa-B; sTRAIL, soluble tumour necrosis factor (TNF)-related apoptosis-inducing ligand; TNF-α, tumour necrosis factor-α; IL-6, interleukin 6; PCT, procalcitonin; PTX-3, pentraxin 3; CRP, C-reactive protein. a: vs control; b: vs breast cancer; * < 0.05; ** < 0.01; *** 0.001.

	Control (n:30)	Breast cancer (n:40)	Colon cancer (n:40)
Age (Year)	48.97±4.95	49.20±7.35	49.88±7.21
Sex (F/M)	15 / 15	40 / 0	20 / 20
BMI (kg/m^2^)	23.49±1.86	25.40±2.95a*	24.57±3.88
CA-15.3 (U/mL)	10.93±3.27	34.54±15.06a***	14.92±5.17^b***^
CA-19.9 (U/mL)	4.47±2.20	7.51±4.08	27.91±8.39^a***,b***^
CEA (ng/mL)	1.49± 0.63	5.83±4.21a**	6.06±7.13^a***^
WBC (x10^9^ /L)	6.65±1.23	7.00±1.66	7.18±2.02
NF-кB (ng/mL)	0.37±0.17	0.98±0.31a***	0.93±0.80^a***^
sTRAIL (pg/mL)	58.93±11.98	50.95±8.26a**	49.60±11.51^a***^
TNF-α (pg/mL)	1.96±0.62	10.79±3.20a***	13.39±3.40^a***,b***^
IL-6 (pg/mL)	4.97±1.89	46.46±23.54a***	64.19±20.12^a***,b***^
PCT (pg/mL)	12.88±3.35	36.02±8.80a***	50.54±13.54^a***,b***^
PTX-3 (pg/mL)	351.53±99.06	1826.25±361.99a***	2208.55±450.44^a***,b***^
CRP (mg/L)	1.63±0.65	4.17±1.55a***	4.71±2.38^a***^

**Table 2:** Clinicopathological features of the patient with breast cancer. ER, estrogen receptor; PR, progesterone receptor

Variables	Breast cancer
	n (%)
No of patients	40 (100)
Menopausal status	
Premenopausal	20 (50)
Postmenopausal	20 (50)
Grade 1/2/3/4	2 (5) / 20 (50) / 14 (35) / 4 (10)
ER - / +	15 (37.5) / 25 (62.5)
PR - / +	21 (52.5) / 19 (47.5)
HER2 - / +	29 (72.5) / 11 (27.5)
Classification	
Luminal	18 (45)
HER2+	11 (27.5)
Triple –	2 (5)
Triple +	9 (22.5)
Metastasis status	
No	23 (57.5)
Yes	17 (42.5)

**Table 3:** Clinicopathological features of the patient with colon cancer

Variables	Colon cancer
	n (%)
No of patients	40 (100)
TNM Stage	
I / II / III / IV	10 (25) / 13 (32.5) / 8 (20) / 9 (22.5)
Tumor size	
≤ 4 cm	21 (52.5)
> 4 cm	19 (47.5)
Metastasis status	
No	25 (62.5)
Yes	15 (37.5)

Correlation analysis between the parameters we tested, tumour markers and the clinicopathological data such as the presence of metastases and the presence of the receptors in the breast cancer and colon cancer patient group was done. According to this, very similarly, NF-κB and TNF-α showed very high positive correlation with tumour grade (respectively r=0.910, p<0.001 and r=0.912, p<0.001), high positive correlation with the presence of HER2 (both r=0.774, p<0.001) and metastasis (respectively r=0.857, p<0.001 and r=0.856, p<0.001), high negative correlation with the presence of ER (both r=-0.839, p<0.001) and PR (respectively r=-0.866, p<0.001 and r=-0.865, p<0.001). On the other hand, sTRAIL, in contrast to NF-κB, showed very high negative correlation with tumour grade (respectively r=-0.914, p<0.001), high negative correlation with the presence of HER2 (r=-0.768, p<0.001) and metastasis (respectively r=-0.858, p<0.001), high positive correlation with the presence of ER (both r=0.840, p<0.001) and PR (respectively r=0.858, p<0.001). IL-6 only had positively low correlation with CA-15.3 (r=0.384, p= 0.014). Correlation tendencies were also in the same direction in the colon cancer group. NF-κB and TNF-α showed very high positive correlation with TNM stage (respectively r=0.965, p<0.001 and r=0.960, p<0.001), high positive correlation with the presence of metastasis (both r=0.839, p<0.001). On the contrary, sTRAIL negatively correlated with TNM stage (r=-0.965, p<0.001) and the presence of metastasis (r=-0.835, p<0.001).

**Figure 1:**
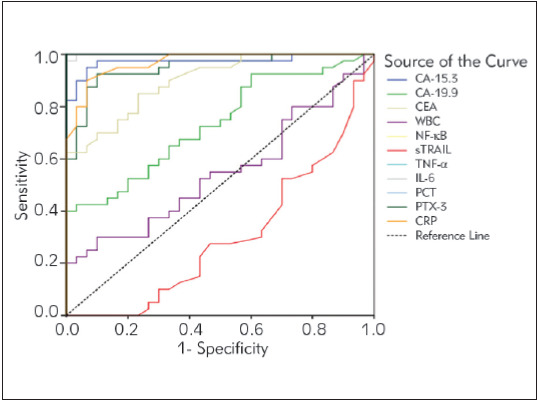
Diagnostic criteria of ROC curve for tested parameters, tumour markers and WBC between breast cancer patients and control groups

Figure 1 summarizes the diagnostic criteria of the ROC curve for tested parameters, tumour markers, and WBC values to differentiate breast cancer from the control subjects. Accordingly, it has been revealed that all parameters except WBC can be used to distinguish breast cancer from control individuals. Risk analysis for breast cancer was performed according to the cut-off points obtained from the ROC curve. It was found that the most increasing parameters of breast cancer risk were high levels of NF-κB, TNF-α, IL-6, PCT, PTX-3, and CRP.

**Figure 2:**
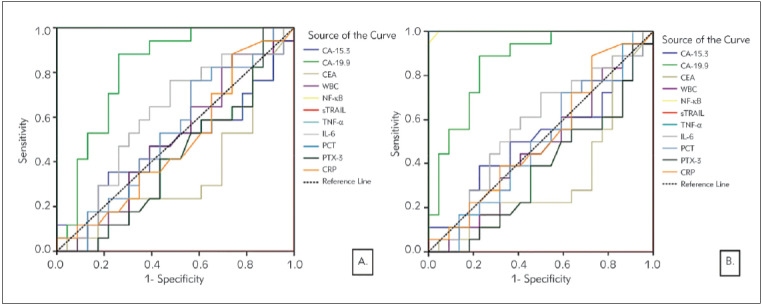
Diagnostic criteria of ROC curve for tested parameters, tumour markers, and WBC. A. Between metastasis and non-metastasis B. Between high-grade (grade 4 and grade 3) and low-intermediate grade (grade 2 and grade 1)

It was also investigated whether the parameters we tested in the breast cancer group could be used to detect metastasis and high grade. When the ROC curves were created for both the presence of metastases (Figure 2A) and (Figure 2B) high grade for the same parameters, it was found that using only NF-κB (AUC= 1.000, p<0.001), TNF-α (AUC= 1.000, p<0.001) and CA-19.9 (AUC= 0.816, p<0.001) would be meaningful for both situations. Therefore, when risk analysis was made only for these three parameters, high levels of NF-κB and TNF-α were found to pose a much greater risk. TNF-α was found to have 100% specificity and sensitivity in distinguishing breast cancer patients with metastases from patients without metastasis (when the cut-off value is selected as 12.33 pg/mL), and high-grade patients from low-intermediate grade patients (when the cutoff value is selected as 12.01 pg/mL). It was found that the specificity and sensitivity of NF-κB in distinguishing metastases was 100% (when the cut-off value is selected as 1.20 ng/mL) and the success of distinguishing high-grade patients had a very high percentage (when the cut-off value is selected as 1.20 ng/mL, Sensitivity: 100%, Specificity: 95%).

**Figure 3:**
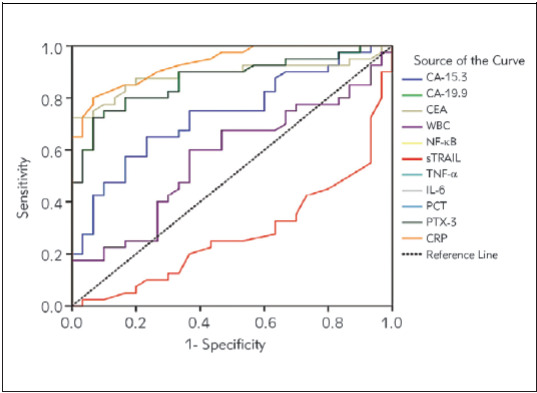
Diagnostic criteria of ROC curve for tested parameters, tumour markers, and WBC between colon cancer patients and control groups

**Figure 4:**
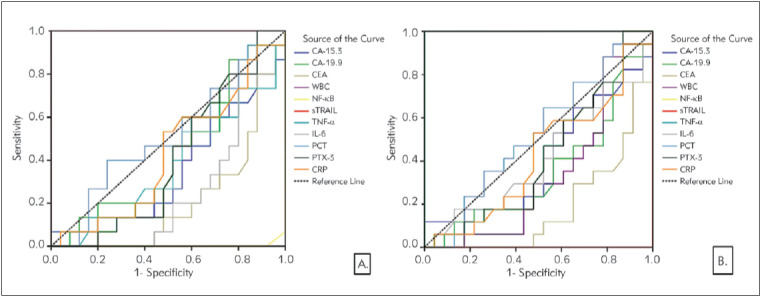
Diagnostic criteria of ROC curve for tested parameters, tumour markers, and WBC in colon cancer patients. A. Between metastasis and non-metastasis B. Between high TNM stage (TNM stage 4 and 3) and low-intermediate stage (TNM stage 2 and 1)

In Figure 3, the results of the analysis of parameters that can be used to distinguish colon cancer from control individuals are given. All parameters except WBC can be used to distinguish colon cancer from control individuals; it was also found that the sensitivity and specificity values of all parameters except sTRAIL were quite high for the determined cut-off values. Risk analysis of the parameters is significant according to the ROC analysis. Accordingly, high values of CA-19.9, TNF-α, PCT, PTX-3, and CRP were found to pose a high risk for colon cancer. In order to understand which of these parameters is a better marker to differentiate the presence of metastases (Figure 4A) and high-grade (Figure 4B) in colon cancer patients, we found that only NF-κBand TNF-α gave significant results when ROC curve, the cut-off values, and risk analysis were performed. For both cases, it was found that the AUC values of NF-κBand TNF-α were 1.000, and both specificity and sensitivity were 100%, and having higher values of NF-κB and TNF-α than the cut-off values significantly increased the risk of metastasis and high grade.

## Discussion

Inflammation is part of the body's response to internal and external environmental stimuli, eliminating incoming stimuli and regulating tissue physiology. Both infectious and non-infectious agents activate inflammatory agents, triggering inflammatory signalling pathways. Inflammatory signalling pathways are usually NF-κB, MAPK, and JAK-STAT pathways. The long duration of these responses causes chronic inflammation and results in many diseases, especially cancer [13]. Clinical and epidemiological studies have shown a strong relationship between chronic infection, inflammation and cancer [14] [15]. NF-κB is involved in cell proliferation, invasion, angiogenesis, and metastases. In this study, we have demonstrated that circulating NF-κB, TNF-α, IL-6, PTX-3, PCT, and CRP levels in breast and colon cancer were significantly higher than in control groups and NF-κB levels were positively correlated with IL-6, PTX-3, PCT, and CRP in all patients. In contrast, sTRAIL levels decreased in these patients, and a negative correlation was found between NF-κB and sTRAIL. Our results indicate that colon and breast cancer patients have increased systemic inflammation, as measured by increased circulating cytokines, and acute-phase proteins, or by abnormalities in circulating cells. NF-κB may combine with other markers of the systemic inflammatory response in prognostic scores. In addition to surgical resection of the tumour, conventional radio- and chemotherapy for cancer treatment, the use of sTRAIL or other agonists, and inhibiting NF-κB signalling has potential therapeutic applications for cancer therapy.

We demonstrated that markedly higher serum IL-6 levels in cancer patients compared to healthy control individuals and a positive correlation between the serum IL-6 levels with the TNF-α and NF-κB levels. IL-6 levels in patients with metastasis were also higher than in patients without metastasis. These findings suggest that IL-6 has strong procarcinogenic activity due to its role in tumour cell proliferation, survival, angiogenesis, inflammation, and metastasis that is one of the activation signals for NF-κB, promoting cell differentiation and subsequent metastasis [16]. Therefore, TNF-α induces pro-inflammatory cytokines such as IL-6 [17] [18], during the inflammatory response [17] [18] [19]. The results of our study show the close link between inflammatory mediators and neoplastic progression and that circulating IL-6 is associated with worse survival in patients with metastatic breast cancer and is correlated with the extent of the disease. Similar to our results, Salgado et al. [20] reported that circulating IL-6 is associated with worse survival in patients with metastatic breast cancer and is correlated with the extent of the disease. The study of Xu et al. [21] confirmed that serum IL-6 might be a potential biomarker for colorectal cancer (CRC) diagnosis, and the high serum IL-6 level was associated with poor prognosis for both CRC overall survival and disease-free survival. Shiga et al. [22] reported that CRC patients with low preoperative levels of IL-6 experienced longer overall survival than those with higher levels of IL-6. But, they did not find a statistical difference in OS rates according to the serum IL-6 level. Chung et al. [23] also demonstrated that tissue expression of IL-6 might also represent a useful predictor of prognosis in CRC [24]. Previous and our studies have shown a relationship between serum IL-6 levels and disease status in colon cancer patients [21] [22] [23] [24] [25] [26].

In the current study, the circulating TNF-α levels in breast and colon cancer were significantly higher than in control groups. NF-κB and TNF-α showed a very high positive correlation with tumour grade, high positive correlation with the presence of HER2 and metastasis, high negative correlation with the presence of ER and PR. When risk analysis was made only for these three parameters, high levels of NF-κB and TNF-α were found to pose much greater risk according to CA-19.9. TNF-α was found to have 100% specificity and sensitivity in distinguishing breast cancer patients with metastases from patients without metastasis (when the cut-off value is selected as 12.33 pg/mL), and high-grade patients from lowintermediate grade patients. Berberoglu et al. [27] suggest that the serum TNF-α levels can be an indicator of response and could be used in clinical decision-making for patients with locally advanced breast cancer. Stanilov et al. [28] demonstrated that serum TNF-α levels in the total group of CRC patients were significantly higher than those in the control group. After grading and staging the cases according to the TNM classification, the highest TNF-α level was found in stage IV of CRC, and it was significantly higher when compared to the earlier stages of CRC and control group. Serum TNF-α was approximately equal among stage I; stage II and stage III of CRC and significantly elevated compared to the controls. Experimental and clinical studies on the role of TNF-α have demonstrated that the TNF-α is a key player in the progression of human breast and colon cancer [27] [28] [29] [30]. We could assume that TNF-α enhances the invasion and metastasis ability of cancer cells via the NF-κB signalling pathway [31].

TRAIL is a transmembrane protein that can also exist in a soluble form, belongs to the TNF superfamily that induces tumour regression and is shown in severe combined immunodeficiency (SCID) mice bearing human tumours, such as colon and breast carcinomas [32] [33] [34]. In the current study, sTRAIL levels were very significantly lower, NF-κB levels were very significantly higher in the patients compared to the control group. There was no statistical variation among the cancer groups when compared to the sTRAIL and NF-κB levels. The circulating sTRAIL is a negative marker for inflammation inversely associated with tumourigenesis in cancer patients. Serum TRAIL levels have been reported in cancer patients in a few previous studies [35] [36] [37] [38]. Contrary to our results, Perik et al. [38] demonstrated that higher plasma levels of both TNF-α and sTRAIL in breast cancer survivors compared to controls. Post-chemotherapy serum TRAIL levels in long-term disease-free breast cancer survivors were higher compared to those in healthy controls. Intensive immune surveillance for cancer cells by high serum TRAIL levels may be required for the long-term survival of advanced breast cancer patients. Interestingly, the serum TRAIL levels were significantly negatively correlated with the serum NF-κB levels in the cancer groups in our study. The relationship between NF-κB and inflammationinduced tumourigenesis was investigated in a metastatic colon cancer mouse model where it was observed that injection with bacterial lipopolysaccharide resulted in metastatic tumour growth via an inflammatory mediator, TNF-α. In vitro, this inhibition with mutant IκBα of NF-κB resulted in a cytocidal effect mediated by TRAIL [39]. NF-κB expression in tumour tissue is associated with angiogenesis and poor 5-year overall survival in stage III colorectal cancer patients [40]. Focusing on cytokine and inflammatory mediators within the whole signalling pathways between TNF-α, TRAIL, also known as Apo2 ligand, and NF-κB provides a comprehensive understanding of this complex pathway as it relates to breast and colon cancer and offers insight into potential therapeutic agents. The NF-κB signalling pathway is important in the carcinogenic process, given its role in the regulation of genes both inside and outside of the immune system. Thus, it can potentially influence many diseases, including CRC [41].

PTX-3, CRP, and PCT levels have been investigated to predict the development of inflammation in various cancers, including breast and colon cancer. We observed that in subjects with breast and colon cancer, the CRP, PTX-3, and PCT levels were significantly and consistently higher than in the control group. There was no statistical variation among the cancer groups when compared to the CRP levels. The PTX-3 and PCT levels were significantly higher in colon cancer compared to breast cancer. Meanwhile, PTX-3 levels were positively correlated with the NF-κB and IL-6 levels in the breast and colon cancer. PTX-3 had the highest sensitivity and specificity. PTX-3 has been suggested to play a significant role in tumour-associated inflammation and was shown to be up-regulated in several malignancies, including melanoma, prostate cancer, breast cancer, and lung cancer [42]. PTX3, a modulator of inflammatory processes, is a member of the highly conserved pentraxin superfamily, which includes CRP, and it is associated with disease severity and mortality in patients with cancer [43] [44]. Up-regulation of PTX-3 gene expression has been described in aggressive breast cancer and distant bone metastases [44] [45] [46] [47]. In CRC, serum PTX-3 levels were significantly increased compared to healthy individuals or patients with colorectal polyps, representing an independent prognostic factor for CRC patients [48]. PTX-3 levels were reduced at discharge after surgery, and a subsequent increase during the follow-up was associated with recurrence. Preoperative PTX3 levels were significantly associated with the clinical stage and with a better postoperative prognosis in a cohort of 263 primary CRC patients [49]. PTX-3 is mainly induced by NF-κB and proinflammatory cytokines, such as IL-6 and TNF-α in breast and colon cancer. The clinical interpretation of elevated PCT concentration in blood represents a great challenge in cancer patients since its values might be influenced by several factors, such as the presence of metastasis or neuroendocrine function of malignant tissue [50]. Some reports claim that PCT can help in differentiating between infection and no infection, but others state that its role is limited and should be interpreted with caution. Increased PTX-3, PCT and CRP levels in the current study, inflammation may be one of the key promoters in the development and progression of cancer.

NF-κB is expressed in the human breast cancer cell lines and in the carcinogenic process of colon cancer that may be an indicator of cancer-associated inflammation as a part of the host immune response. In addition to CRP, PTX-3, PCT or in combination with clinical parameters should be considered in routine measurement to identify critical patients with inflammation as early as possible. The circulating IL-6, TNF-α, TRAIL level in breast and colon cancer may be a biomarker of postoperative progression or recurrence. Observations from this study further support the link between NF-κB-induced inflammation and the growth of malignant cells in breast and colon cancer.

## Acknowledgments

This research did not receive any specific grant from any funding agency in the public commercial or a non-profit section.

## Conflict of interest statement

The authors stated that they have no conflicts of interest regarding the publication of this article.
